# A field experiment on the effects of weekly planning behaviour on work engagement, unfinished tasks, rumination, and cognitive flexibility

**DOI:** 10.1111/joop.12430

**Published:** 2023-02-17

**Authors:** Lars Uhlig, Vera Baumgartner, Roman Prem, Katja Siestrup, Christian Korunka, Bettina Kubicek

**Affiliations:** ^1^ Institute of Psychology University of Graz Graz Austria; ^2^ Faculty of Psychology University of Vienna Vienna Austria; ^3^ Department of Work and Organizational Psychology, Faculty of Psychology FernUniversität in Hagen Hagen Germany

**Keywords:** cognitive flexibility, field experiment, planning behaviour, rumination, unfinished tasks, work engagement

## Abstract

This study concerns research on self‐regulation. It examines the effects of planning behaviour, a comprehensive self‐regulatory strategy of goal setting, planning work steps, and developing alternative plans. Combining different strategies, rather than testing them in isolation, would strengthen their effects and make them more appropriate for complex work tasks. Drawing on self‐regulation theory, we propose that planning behaviour positively affects work engagement, unfinished tasks, rumination, and cognitive flexibility. Considering cognitive flexibility as an outcome provides insight into the cognitive benefits of planning behaviour. We examine person‐level cognitive demands of flexible work and predictability as moderator variables to better understand the role of contextual variables in the use of self‐regulatory strategies at work. We conducted a field experiment (*N* = 208 individuals; 947 weekly entries) in which we manipulated employees' weekly planning behaviour in their daily work lives. We found negative effects on unfinished tasks and weekly rumination, and positive effects on weekly cognitive flexibility. No significant moderating effects were found. Our study suggests that a brief planning manipulation at the beginning of the week may have multiple benefits and may be an important tool for improving cognitive flexibility. Future research should examine the role of time and mediating variables.

**Practitioner points**

- Weekly planning behaviour, which includes goal setting, planning work steps, considering potential obstacles, and developing alternative plans, shows positive effects on unfinished tasks, after‐work rumination, and cognitive flexibility in employees.
- Encouraging planning behaviour, especially considering obstacles and developing alternative plans, could be an important tool to improve perspective taking, adaptability, and problem solving.
- Planning behaviour can be supported by providing specific and prioritized goals and time management tools, and by ensuring the availability of necessary information. Encouraging people to reflect on the accuracy of their plans should improve the quality of planning behaviour.

## BACKGROUND

Many employees today face challenging work environments that are highly dynamic and often involve working on open‐ended and ill‐defined tasks (Grant & Parker, 2009). Organizations expect high levels of work engagement (Grant & Parker, 2009; Kanfer et al., 2017), which is often harmed by frequent interruptions and obstacles that prevent timely task completion and require high levels of flexibility from employees (Allvin et al., 2011; Jett & George, 2003; Parke et al., 2018). Due to high demands, employees may experience after‐work rumination, which may limit recovery between workdays (Bennett et al., 2016; Dettmers & Bredehöft, 2020).

Self‐regulation strategies such as prioritizing and goal setting, planning work steps, anticipating potential obstacles, and developing alternative plans can help employees cope with these challenges (Austin & Vancouver, 1996; Frese & Zapf, 1994; Lord et al., 2010; Mumford et al., 2001). Goal setting and planning work steps are known to increase goal commitment, help people get started on the task, and reduce the risk of getting derailed in the pursuit of goals (Austin & Vancouver, 1996; Frese & Zapf, 1994; Gollwitzer & Sheeran, 2006; Mumford et al., 2001). Specifying when, where, and how a task will be completed can automate future actions, free up attentional resources, and reduce the ambiguity associated with a task (Gollwitzer & Sheeran, 2006; Masicampo & Baumeister, 2011; Smit, 2016; Smit & Barber, 2016). Moreover, thinking about potential obstacles and developing alternative plans can provide cognitive benefits that help employees to respond flexibly in the face of changing circumstances (Frese & Zapf, 1994; Mumford et al., 2001; Parke et al., 2018; Tripoli, 1998). Therefore, self‐regulation strategies could be a valuable tool in addressing the challenges associated with contemporary workplaces.

To date, most studies have focused on one self‐regulatory strategy at a time, such as prioritizing and goal setting (Locke & Latham, 2002) or planning work steps (Gollwitzer, 1999). This approach works well for relatively simple actions and goals, but more sophisticated strategies are needed to accomplish complex tasks in dynamic work environments (Frese & Zapf, 1994; Lord et al., 2010; Mumford et al., 2001). Self‐regulation should be more effective when strategies are combined, as each strategy brings different benefits that may complement each other. For example, planning work steps is thought to enhance the positive effects of goal setting on goal attainment because it details how, when, and where a goal can be achieved, making it easier to act on opportunities as they arise (Gollwitzer & Sheeran, 2006). Furthermore, considering potential obstacles and developing alternative plans after planning work steps can help avoid overly optimistic planning and frustration (Buehler et al., 1994; Frese & Zapf, 1994; Parke et al., 2018).

In this study, we sought to investigate the effects of a comprehensive self‐regulation strategy. We subsume different self‐regulation strategies under the umbrella term “planning behaviour,” which we define as a complete planning process of future actions aimed at achieving a particular work goal (Frese & Zapf, 1994; Mumford et al., 2001). This would include prioritizing and setting goals, identifying and planning work steps, considering potential obstacles, and developing alternative plans.

Drawing on self‐regulation theory, we propose that engaging in planning behaviour at the beginning of the week has beneficial effects on weekly work engagement, unfinished tasks at the end of the week, weekly after‐work rumination, and weekly cognitive flexibility. The beneficial effects of self‐regulatory strategies on engagement and task performance are central propositions of self‐regulation theory and are thought to result from facilitating both the initiation and maintenance of action (Austin & Vancouver, 1996; Gollwitzer & Sheeran, 2006; Locke & Latham, 2002; Parke et al., 2018). Scholars have built on self‐regulation theory to show that planning and specifying work steps can reduce employees' rumination by clarifying how a particular task will be completed and providing cognitive resolution (Masicampo & Baumeister, 2011; Smit, 2016; Smit & Barber, 2016). Finally, self‐regulation theory suggests that planning behaviour can increase flexibility in thinking as it leads to more thorough searching and information processing to gain a better understanding of a task (Frese et al., 2007; Frese & Zapf, 1994; Giorgini & Mumford, 2013).

We test these effects by manipulating weekly planning behaviour in a naturalistic field setting using a weekly diary design. Our study will thus advance understanding of the effects of a combined self‐regulatory strategy. Specifically, subsuming goal setting, planning work steps, and anticipating obstacles into a single planning manipulation allows us to test the effects of a work strategy that is tailored to address the challenges of contemporary work (Frese & Zapf, 1994; Mumford et al., 2015; Parke et al., 2018). In addition, manipulating planning behaviour in real work settings allows us to overcome the methodological limitations of previous studies on work‐related self‐regulation, which either lacked ecological validity, for example, were laboratory studies (Diefendorff & Lord, 2003; Masicampo & Baumeister, 2011), or were inconclusive regarding the direction of cause and effect (Claessens et al., 2004, 2007; Parke et al., 2018; Tripoli, 1998).

Examining the effects on cognitive flexibility allows us to contribute to the ongoing debate on whether planning behaviour can improve employees' flexibility and adaptability (Bieleke et al., 2018; Diefendorff & Lord, 2003; Gollwitzer et al., 2008; Mumford et al., 2001). Planning helps employees develop a deeper and more elaborate understanding of the task, the actions required to complete it, and potential obstacles along the way. An improved mental model could make employees more cognitively flexible during goal pursuit (Frese et al., 2007; Frese & Zapf, 1994; Mumford et al., 2001). To date, there has been little empirical research on this topic. Investigating the effects of planning behaviour on cognitive flexibility would advance knowledge about the specific ways in which planning behaviour benefits employee performance. Such knowledge is of considerable practical relevance, as cognitive flexibility is not only crucial for adapting to changing environments but also an important part of problem solving. As such, it represents an essential skill in the contemporary world of work (Grant & Parker, 2009; Rios et al., 2020).

When planning behaviour is tested outside a controlled environment and applied in a real work setting, it becomes important to examine relevant contextual variables (Lord et al., 2010; Rousseau & Fried, 2001). We propose that planning behaviour is more beneficial when it matches the demands of an individual's job. More specifically, we argue that the benefits of planning behaviour are more pronounced for individuals whose jobs have high cognitive demands of flexible work and low predictability. Testing person‐level cognitive demands of flexible work and low predictability as moderator variables will advance our understanding of the relevant boundary conditions for planning behaviour at work (Lord et al., 2010). It will also allow us to identify strategies for coping with these demands and inform decisions about which group planning behaviour should be recommended.

In conclusion, this study will make three contributions to the literature. First, we will apply a combined approach to self‐regulation strategies and examine the effects of a strategy that involves a complete planning process at work. This will teach us how to integrate different self‐regulation strategies to simultaneously address different challenges of contemporary work life, in particular how to increase work engagement, work effectiveness (as indicated by fewer unfinished tasks) and cognitive flexibility, and reduce rumination. Manipulating planning behaviour in a naturalistic field setting represents an important methodological advance and could provide a low‐cost intervention strategy to improve the quality of employees' working lives. Second, examining the effects of planning behaviour on cognitive flexibility will increase knowledge about the potential benefits of planning behaviour. Our study will thus contribute to both theoretical and practical discussions about whether planning behaviour can make employees more adaptable in the face of challenges. Third, our study will examine the cognitive demands of flexible work and predictability as person‐related moderator variables to advance knowledge about the specific conditions under which planning behaviour is most valuable. This will add the necessary nuance to self‐regulation theory, which is crucial if it is to be applied in the work context (Lord et al., 2010).

### Theoretical background

We draw on self‐regulation theory to explain the details of planning behaviour and its potential benefits for employees' work engagement, unfinished tasks, rumination, and cognitive flexibility. Self‐regulation theory is a theoretical framework that describes and explains the motivational, cognitive, and affective processes that drive and influence goal‐directed behaviour (Lord et al., 2010; Vancouver & Day, 2005). A core concept in self‐regulation theory concerns goals, which are internal representations of desired future outcomes that guide individuals through action and serve as an internal standard against which they compare their current state (Austin & Vancouver, 1996; Frese & Zapf, 1994). Individuals often redefine their goals according to their attitudes, values, and expectations, and they usually have some freedom in how they formulate their goals, for example, they can be more or less specific (Austin & Vancouver, 1996; Frese & Zapf, 1994). As employees often have several goals at the same time, it is necessary to prioritize them (Gollwitzer & Sheeran, 2006). Goals that are specific and challenging are most effective in guiding and encouraging individual effort (Gollwitzer, 1990; Locke & Latham, 2002).

In addition to prioritizing and setting goals, planning work steps plays a crucial role in self‐regulation theory. This involves identifying the work steps that are essential to achieving a goal and organizing them in a practical way. This clarifies which work steps are necessary and guides the individual through the action process (Claessens et al., 2007; Mumford et al., 2001). Work steps should be contextualized, that is, the place, time, and possibly the people with whom it is necessary to collaborate should be defined (Gollwitzer, 1996; McCrea et al., 2008; Mumford et al., 2001). Specifying the context increases the likelihood that work steps will actually be performed by automating future actions (Diefendorff & Lord, 2003; Gollwitzer & Sheeran, 2006; McCrea et al., 2008). It allows individuals to allocate their resources more efficiently and to identify and avoid conflicts when allocating the necessary resources for each work step (Mumford et al., 2001).

Self‐regulation theory suggests that obstacles should be anticipated and alternative plans developed (Frese & Zapf, 1994; Mumford et al., 2001; Parke et al., 2018; Tripoli, 1998). Individuals may face interruptions (Jett & George, 2003), external barriers (Frese & Zapf, 1994; Mumford et al., 2001), or internal barriers such as loss of motivation or procrastination (Steel & Weinhardt, 2018). Developing alternative plans can improve the quality of the plan, as it draws attention to potential problems and weaknesses and allows one to adapt and prepare accordingly (Giorgini & Mumford, 2013).

#### Effects of planning behaviour on work engagement

Work engagement describes a “positive, fulfilling, work‐related state of mind” (Schaufeli & Bakker, 2004), characterized by a high willingness to invest effort, experiences of immersion in a task, and feelings of enthusiasm (Schaufeli & Bakker, 2004). Planning behaviour can benefit from these states, as prioritization and goal setting mobilize effort and increase goal commitment (Locke & Latham, 2002; Nenkov & Gollwitzer, 2012). Furthermore, having a specific goal and a detailed plan can focus the employee's attention on the work that remains to be done to achieve the goal and motivate them to invest further effort (Johnson et al., 2006; Locke & Latham, 2002).

Immersion in work may become more likely as planning behaviour facilitates action. When employees plan the necessary work steps and understand their context, initiating, and sustaining actions can become automatic (Gollwitzer, 1999; Gollwitzer & Sheeran, 2006). This means that employees do not have to think about their next work step, but can focus on the action itself. Planning behaviour can also support immersion by providing employees with alternative plans in the face of complications and guidance on how to deal with the situation, thereby helping employees to remain focused (Gollwitzer, 1999; Gollwitzer & Sheeran, 2006; Parke et al., 2018).

Finally, planning can help employees develop an enthusiasm for their work. Enthusiasm is often dampened by problems or obstacles (Jett & George, 2003), and planning behaviour helps employees to mentally prepare for such obstacles by providing a concrete plan for overcoming them (Frese & Zapf, 1994; Parke et al., 2018; Tripoli, 1998). This should not only reduce frustration in the face of obstacles but also generate positive emotions when problems are successfully overcome (Bledow et al., 2011).

Thus, we expect planning behaviour to be associated with higher work engagement during the week.

Hypothesis 1 Weekly planning behavior is positively associated with weekly work engagement.

#### Effects of planning behaviour on unfinished tasks

As explained above, setting specific and challenging goals and planning how to achieve them has various beneficial effects on the effort that individuals invest (Gollwitzer, 1999; Johnson et al., 2013; Locke & Latham, 2002), resulting in greater effectiveness and a higher probability of completing tasks. Planning behaviour allows resources to be allocated more efficiently and reduces the likelihood of being derailed by obstacles (Frese et al., 2007; Gollwitzer & Sheeran, 2006; Maisto et al., 2015; Parke et al., 2018; Tripoli, 1998). Furthermore, studies show the positive effects of planning behaviour on task performance (Claessens et al., 2007; Diefendorff & Lord, 2003; Frese et al., 2007; Parke et al., 2018; Tripoli, 1998). Therefore, we expect that planning behaviour will lead to fewer unfinished tasks at the end of the week.

Hypothesis 2 Weekly planning behavior is negatively associated with weekly unfinished tasks.

#### Effects of planning behaviour on rumination

After‐work rumination refers to persistent, repetitive thoughts about work during leisure time (Martin & Tesser, 1996). As these thoughts are often accompanied by negative feelings, rumination can be distressing (Cropley et al., 2012; Frone, 2015). Planning behaviour could help reduce rumination (Masicampo & Baumeister, 2011; Smit, 2016; Smit & Barber, 2016). Thinking through the necessary work steps and writing down each step could reduce employees' concerns about forgetting tasks or work steps. The Zeigarnik effect may also play an important role. This refers to the finding that unfinished tasks tend to be more cognitively available to individuals (Zeigarnik, 1927, 1938). In planning behaviour, tasks are broken down into smaller steps that can be ticked off over the course of the week. Therefore, planning behaviour may allow employees to experience cognitive resolution and make work less cognitively available when employees are not working (Johnson et al., 2006; Syrek et al., 2017). Finally, research on emotion regulation strategies suggests that planning may reduce rumination because individuals feel better prepared to actively deal with upcoming challenges (Sacchi & Dan‐Glauser, 2021; Zlomke & Hahn, 2010). Thus, we expect that planning behaviour will reduce rumination in employees during the week.

Hypothesis 3 Weekly planning behavior is negatively associated with weekly rumination.

#### Effects of planning behaviour on cognitive flexibility

Cognitive flexibility refers to the ability to adapt one's cognition and behaviour to changing situations. It requires an active problem‐solving style and the ability to generate multiple approaches to solving problems, to consider multiple perspectives (including those of others), and to modify one's strategy or plan when necessary (Dennis & Vander Wal, 2010; Krems, 1995). It shows some inter‐individual trait‐like stability (Ionescu, 2012); however, cognitive flexibility varies within individuals: individuals may be more or less cognitively flexible in different situations (e.g., Isen, 2002).

Planning behaviour could increase cognitive flexibility because it initiates the search for and processing of information, activates necessary knowledge, and involves cognitive (re)structuring of tasks (Frese et al., 2007; Locke & Latham, 2002; Mumford et al., 2001; Wood et al., 2013). This can lead to more organized mental representations of the action, allowing important issues and potential problems to become more salient to the individual (Frese et al., 2007; Frese & Zapf, 1994; Mumford et al., 2001). This type of mental representation could benefit one's cognitive flexibility by enhancing coping and adaptability and generating new approaches and perspectives. Being aware of key issues and potential problems could improve individuals' processing of relevant information, allowing employees to respond more quickly and proactively to emerging opportunities or challenges (Frese & Zapf, 1994; Presbitero, 2015; Wood et al., 2013). Thus, planning behaviour encourages individuals to generate new ideas and multiple approaches to solving problems (Giorgini & Mumford, 2013; Montani et al., 2015). Therefore, employees may be more likely to consider the perspectives and motivations of others, which are often important for predicting potential problems and solutions (Parker et al., 2008). Thus, we expect planning behaviour to be associated with higher cognitive flexibility during the week.

Hypothesis 4 Weekly planning behavior will be positively associated with weekly cognitive flexibility.

#### The role of cognitive demands of flexible work

Planning behaviour needs to match the demands that people face in their jobs. It can only be useful in jobs that require a certain amount of forward thinking (Frese & Zapf, 1994). When tasks are highly predetermined, planning behaviour becomes pointless as there is little room for its beneficial effects to unfold. Cognitive demands of flexible work fit well with planning behaviour. In flexible work environments, employees need to plan when and where they will work and on what tasks, as the time and place of work are not always fixed (Allvin et al., 2011; Prem et al., 2021). As most work tasks in flexible work environments involve collaboration, employees need to consider how and when to coordinate with others, which often involves planning ahead. Plans are often necessary for collaboration, as specifying goals and necessary work steps provide a useful basis for communicating a common approach in a team (Mumford et al., 2001). Further, employees in flexible work environments are required to structure their work tasks independently. They have to plan the necessary work steps, determine the sequence of these steps, and monitor their own progress (Allvin et al., 2011; Prem et al., 2021). Planning is thus central to managing the high cognitive demands of flexible work (Allvin et al., 2011; Mohr & Wolfram, 2010; Prem et al., 2021). Consequently, we expect that the positive effects of planning behaviour should be more pronounced when employees work in jobs with high cognitive demands of flexible work.

Hypothesis 5 The relationships between weekly planning behavior and weekly work engagement, cognitive flexibility, rumination, and unfinished tasks will be moderated by planning working times, planning work places, structuring work tasks, and coordinating with others. These relationships will be stronger for employees with high levels of these demands compared to employees with low levels of these demands.

#### The role of predictability

Building on the work of Mohr and Wolfram (2010), Schoellbauer et al. (2022, p. 3) define low predictability at work as a state in which employees have difficulty anticipating “which work‐related tasks and activities they will have to perform on any given day, including methods, time requirements, and potential problems that may arise in the process.” Many of the benefits of planning behaviour are enhanced under such conditions (Mumford et al., 2001). For example, planning behaviour triggers and intensifies the search for new and relevant information (Frese & Zapf, 1994; Mumford et al., 2001; Wood et al., 2013). This type of active search is more important under conditions of low predictability, as information is less readily available (Mohr & Wolfram, 2010; Schoellbauer et al., 2022). Further, having a ready‐made plan can help people regain goal focus when other tasks or demands have distracted them because the plan provides clear instructions on how to act to regain goal focus (Gollwitzer & Sheeran, 2006). In conditions of low predictability, this advantage would come into play more often, as people face more distractions (Jett & George, 2003; Schoellbauer et al., 2022). Similarly, alternative plans may come into play more often in conditions of low predictability, as obstacles are more frequent (Mohr & Wolfram, 2010; Parke et al., 2018; Schoellbauer et al., 2022). Having developed an alternative plan is useful even when an obstacle is not predicted because employees have already considered alternative routes to reach their goal. Consequently, employees should adapt more quickly to changing situations and find ways to overcome unexpected obstacles (Mumford et al., 2001; Parke et al., 2018; Wood et al., 2013). Therefore, employees working in jobs with low predictability should derive more benefits from planning behaviour.

Hypothesis 6 The relationships between weekly planning behavior and weekly work engagement, cognitive flexibility, rumination, and unfinished tasks will be moderated by person‐level predictability. The relationships will be stronger for employees with low levels of predictability compared to employees with high levels of predictability.

**FIGURE 1:**
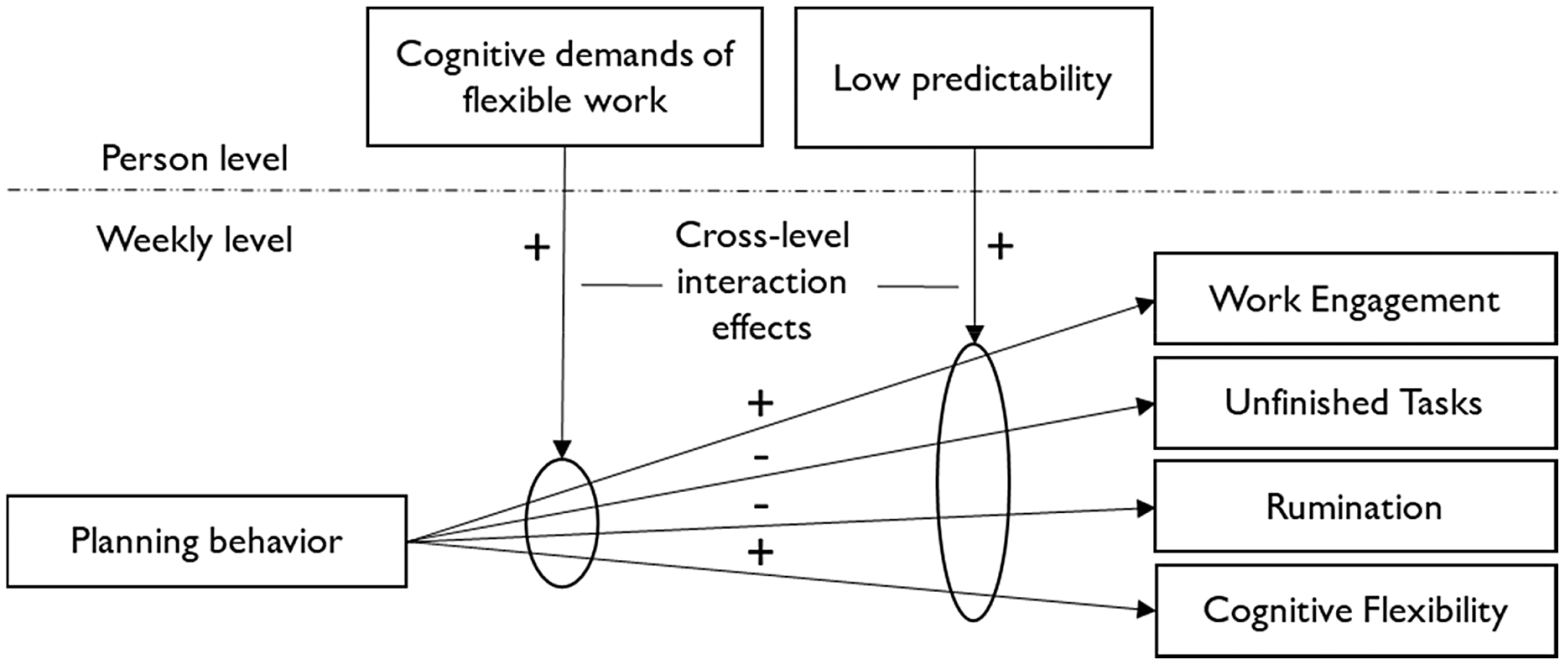
Conceptual model.

A depiction of the conceptual model of the study is outlined in Figure 1.

## METHOD

### Study design

**FIGURE 2:**
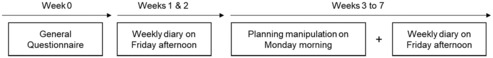
Depiction of the study design.

This study uses a within‐subject design, which means that all participants were tested in both conditions: with and without planning manipulation. This design allows researchers to control for individual differences in the participants' overall levels on the four outcomes and has a higher power to detect the hypothesized effects (West et al., 2008). The study design is outlined in Figure 2. We used a general questionnaire that investigated person‐levels of cognitive demands of flexible work and predictability; participants completed the questionnaire when enrolling in the study. All participants experienced 2 weeks without planning manipulation followed by 5 weeks with planning manipulation, and we collected data on the four outcome variables for both conditions on Friday afternoons. The planning manipulation was completed on Monday mornings. To remind participants about their goals and plan for the week, during weeks three through seven participants were asked to report on their goal progress on Wednesday afternoons.

### Procedure

An institutional review board on human subject research at a public university in Austria approved our data collection protocol (2019/A/001). Throughout 2019, using multiple strategies to recruit participants, we collected study data. First, we invited people to participate in the study via an email newsletter sent out by a company specializing in corporate health management. Second, we asked an IT company's human resources department to invite employees (via email) to participate in the study. Employees were offered the incentive of winning one vacation day in a raffle if they participated in the study for at least 5 weeks. Third, we asked psychology students at two universities, one in Germany and one in Austria, to recruit participants or to participate themselves in exchange for course credits. These students received clear and detailed information on the procedure and the requirements for participation. They were only invited to participate if they were employed and studying part‐time. The course credits that could be collected per student were limited. We provided comprehensive information on the study's procedure and the planning manipulation; the study was described as training for new ways to plan and manage weekly work tasks. To increase compliance, all participants were offered individual feedback on their weekly responses after the study was completed.

During study recruitment, participants were asked to only enrol in the study if they worked in a flexible work regime, such as having flexible working hours in a teleworking arrangement or working regularly on different projects with changing teams. This inclusion criterion ensured that participants had sufficiently complex work with changing working conditions such as different hours, working places, tasks, or teams, so that weekly planning would be advantageous and non‐redundant. Participants were also required to work at least 20 hours per week. All participants completed an informed consent on enrollment in the study.

### Planning manipulation

The planning manipulation consisted of four parts: prioritizing and goal setting, planning work steps, planning for hindrances, and visualization. The participants were guided through a dynamic online form to complete all four parts. The median time the participants took to complete the planning manipulation was 7.9 min.

#### Prioritizing and goal setting

The first part of the planning manipulation focused on improving prioritizing and goal setting (Locke & Latham, 2002, 2013). The participants were asked to provide three work goals they would like to achieve by the end of the week and to rank them by importance. They were then asked to reformulate the most important goal, which had to be specific, measurable, and challenging, but realistic; we also offered an example of how a goal could be reformulated so as to satisfy these criteria. Participants typically formulated their goals in short, abbreviated ways—for example, “prepare a PowerPoint of all observations and explanations,” “make a mind map with all main and secondary objectives,” “prepare all bills from 01.05 till 20.05. until 25.05,” or “finish analysis for four main categories and targets.”

#### Planning work steps

Participants were asked to define the work steps necessary to reach their reformulated goals and define situational cues to execute these work steps (Gollwitzer & Sheeran, 2006). They were then asked to define a deadline for finishing each work step and, where appropriate, to name the people with whom they will work on each step. They also defined the location at which each step was to be performed. For example, one participant planned four work steps for their goal to “prepare a PowerPoint of all observations and explanations”: work step 1, “analyze how BA is now used,” with a deadline of “Tuesday”; work step 2, “understand the file from Germany,” with a deadline of “Tuesday”; work step 3, “ask Selin and Peter about the use of BA,” with a deadline of “Wednesday”; and work step 4, “build a PPT,” with a deadline of “Thursday.”

#### Planning for hindrances

We asked participants to identify a potential hindrance for each work step and define a plan to address this hindrance (Parke et al., 2018; Tripoli, 1998); we advised them to formulate the plan according to an implementation intention (Gollwitzer, 1999). Implementation intentions are “if‐then” plans in which a specific situation is defined (if‐part) that is coupled with the initiation of a specific behaviour (then‐part). In this case, the *if‐part* contained the potential obstacle, and the *then‐part* contained the contingency plan through which the obstacle could be overcome. An example of this type of implementation intention is: “If I feel distracted during my work, I will close the door to my office and block time in my calendar.” In an example from our data, the participant described above who was pursuing their weekly goal to “prepare a PowerPoint of all observations and explanations” formulated the following alternative plans for their work steps 1–4: “If I don't understand how the file is structured, then I will ask a colleague to help me”; “If I don't understand how the Germans work, then I will ask a colleague”; “If neither is available, then I will ask Sandra”; and “If I did not gather enough information, then I will ask for more information from colleagues.”

#### Visualization

Finally, we asked participants to visualize a situation in which the predicted obstacle arises for each of their work steps and how they would act according to their contingency plan. Mental imagery techniques such as these have been found to improve the probability that plans, such as implementation intentions, are put into practice (Knäuper et al., 2009; Loft & Cameron, 2013).

### Participants

In total, 231 participants provided 1104 completed weekly entries. We checked all weekly plans and excluded planning manipulation data in which participants did not set a goal for their week (e.g., filled in “x” or left a blank) or did not plan at least one work step. This led to the exclusion of 25 weekly entries and five participants. Some participants filled out the planning manipulation to plan goals that were related to their leisure time (e.g., they planned their vacations) or their studies (e.g., they planned their study times for an exam). Since the manipulation was intended to change planning behaviour regarding work tasks and all of the outcomes were work‐related, we discarded data for which the weekly plans were not related to work tasks. This led to the exclusion of 157 weekly entries and 18 participants. The final sample consisted of 208 participants with 947 weekly entries. On average, participants provided 5.8 weeks' worth of data with an average of 1.9 weeks without the planning manipulation and 4.0 weeks with the planning manipulation.

Of the final sample, 40 participants (19.2%) were recruited via newsletter, 43 participants (20.7%) were recruited via the IT company, and 125 participants (60.1%) were recruited either by students or were themselves students. Following Demerouti and Rispens' (2014) recommendations, we compared the student‐recruited sample with a merged sample of participants recruited via the newsletter and via the IT company to examine whether the student‐recruited sample differed with regard to socio‐demographic characteristics. We found no significant differences in the subsamples with regard to educational level, leadership position, reported planning behaviour, dependent variables, or moderators. However, the participants recruited via students had a significantly higher age (*M* = 42.00, *SD* = 11.63) than participants of the sample recruited via the newsletter and the IT company (*M* = 34.43, *SD* = 10.49).^1^

The age of participants averaged 37.08 years (*SD* = 10.69), with an average tenure of 4.80 years (*SD* = 5.18). Of the sample, 61.0% were female; 127 participants (63.2%) reported having no leadership position, 74 participants (36.9%) reported having a position in middle management or higher; 116 participants (57.7%) had a university degree, 52 participants (25.9%) had a high‐school diploma, and 33 participants (16.5%) had finished at least an apprenticeship. Participants worked, on average, 38.35 hours per week (*SD* = 10.94). Their occupations were diverse, ranging from software development to consultancy to social work. Participants worked in a range of sectors, including the service sector (54.5%), the education sector (10.3%), the industrial sector (10.2%), and the health care sector (9.2%).

### Instruments

All answers were rated on a 7‐point Likert scale ranging from 1 (“do not agree at all”) to 7 (“fully agree”). We calculated within‐person alphas and between‐person alphas following Geldhof et al.'s (2014) approach.

The general questionnaire included questions regarding the cognitive demands of flexible work and predictability in the participants' jobs; we presented it to participants immediately upon enrollment in the study. To adapt the questionnaire items to refer to the preceding 5 weeks, we added “over the last five weeks” to each item; we chose this time range for data collection to ensure a current assessment of the participants' general work characteristics at the time of their enrollment. Gathering data on current work characteristics allowed us to minimize recall bias and increase the relevance of reported work characteristics for participants' momentary experiences (Walentynowicz et al., 2018). Measuring the cognitive demands of flexible work and predictability before participants engaged in the planning manipulation ensured that the planning manipulation did not influence their perceptions of those demands. Furthermore, we reduced common method bias by measuring the moderator variables and outcome variables at separate points in time (Podsakoff et al., 2012).

We adapted Prem et al.'s (2021) scale to measure the cognitive demands of flexible work. This scale comprises four subscales: coordinating with others, structuring work tasks, planning working times, and planning working places.

We used three items that referred to demands to coordinate with others at work. A sample item is, “Over the last five weeks, my job required me to come to an agreement with other people regarding a common approach.” The between‐person alpha for this scale was .86.

We used three items that investigated the degree to which participants had to structure and monitor their work tasks self‐reliantly in their job to measure structuring of work tasks. A sample item is, “Over the last five weeks, my job required me to determine the sequence of my work steps on my own.” The between‐person alpha for this scale was .84.

We used three items that asked whether participants decided on or planned their own working times to measure planning of working times. A sample item is, “Over the last five weeks, I had to decide how long I work on which weekdays due to my flexible schedule.” The between‐person alpha for this scale was .85.

We used three items that examined whether participants had to plan where to work to measure planning of working places. A sample item is, “Over the last five weeks I had to plan where to work on certain tasks, because I do not have the same work materials available everywhere.” The between‐person alpha for this scale was .88.

We used four adapted items from Schyns and Paul's (2004, as cited in Mohr & Wolfram, 2010) scale to measure predictability. This measure identified whether upcoming tasks, problems, and solutions had been predictable at work. A sample item is, “Over the last five weeks, I could predict which problems I would encounter when completing my tasks.” The between‐person alpha for this scale was .76.

Participants completed weekly questionnaires on Friday afternoons. We adapted all weekly measures except the unfinished tasks measure by adding “this week” to all items.

We used the ultra‐short version of the Utrecht Work Engagement Scale (UWES‐3; Schaufeli et al., 2019) to measure work engagement. This scale consists of three items, including one item each to measure willingness to invest effort, experiences of immersion, and feelings of enthusiasm. A sample item is, “This week, I felt bursting with energy at my work.” The within‐person alpha for this scale was .74; the between‐person alpha was .85.

We measured unfinished tasks with three items from Syrek et al.'s (2017) scale. This scale assesses whether important or urgent tasks were finished during the work week. A sample item is, “I have not finished important tasks that I had planned to do this week.” The within‐person alpha for this scale was .87; the between‐person alpha was .97.

We measured rumination using three items from Frone's (2015) negative rumination scale. This measure assesses negative repetitive thoughts about work. A sample item is, “This week, I kept thinking about the negative things that happened at work even when I was away from work.” The within‐person alpha for this scale was .90; the between‐person alpha was .99.

We used four items from Dennis and Vander Wal's (2010) scale to measure cognitive flexibility. These items investigated whether participants had considered different perspectives (including the perspectives of others) in situations they encountered. A sample item is, “This week, I tried to think about things from another person's point of view.” The within‐person alpha for this scale was .80; the between‐person alpha was .98.

### Manipulation check

We used a manipulation check to assess whether the planning manipulation led to more planning behaviour. We used adapted and shortened scales measuring time‐management planning and contingency planning (Parke et al., 2018) to measure planning behaviour on Friday afternoons throughout the 7 weeks of the study. We shortened the scales to three items each and adapted them by adding the prefix “this week” to all items. The items for time‐management planning examined whether participants had engaged in time‐management planning behaviour, such as prioritizing, planning, and time allocation. An example item is, “This week, I have set priorities for my tasks.” The within‐person alpha for this scale was .65; the between‐person alpha was .88. The contingency planning scale asked whether participants had considered potential hindrances or obstacles and had made plans to overcome them. An example item is, “This week, I have developed alternative courses of action in case my tasks are interrupted or disrupted.” The within‐person alpha for this scale was .81; the between‐person alpha was .92.

### Analysis

The data were hierarchically structured; week‐level data were nested in person‐level data. To account for this, we tested the hypotheses using multilevel modelling. To test hypotheses 1 through 4, we regressed work engagement, unfinished tasks, rumination, and cognitive flexibility on a dichotomous variable representing the planning manipulation (no/yes). The effects were specified on the within‐person level with random slopes for each effect. To reduce the complexity of the model and facilitate the convergence of the model, random slopes were set to be uncorrelated. The variable representing the planning manipulation was group‐mean centered before the analysis. We conducted the analyses with Mplus version 8.5, using a Bayesian estimator with default non‐informative priors and means for point estimates. Bayesian estimation for multilevel models with random slopes provides a straightforward way to calculate the total variance explained in the within‐person level *R* ^2^ as indicators of effect sizes (LaHuis et al., 2014; Schuurman et al., 2016). Moreover, it does not rely on the normality assumption. However, we only used Bayesian estimation as a computational tool—we did not apply a full Bayesian inference approach. Since we used non‐informative priors, the study's conclusions are not affected when using maximum‐likelihood estimation.

Bayesian estimation in Mplus uses Markov chain Monte Carlo (MCMC) algorithms. A potential scale reduction of <1.10 for all parameters is used as the default criterion for convergence of the MCMC sequence in Mplus. Three MCMC chains were used and the first half of the iterations were used as burn‐in. To evaluate convergence, we visually inspected trace and autocorrelation plots for all estimated parameters (Kaplan & Depaoli, 2012).

For all outcomes, we have reported within‐level *R* ^2^, which refers to the total variance explained in the outcome variables on the within‐person level averaged across all persons (Schuurman et al., 2016). This indicates how much variance the planning manipulation explained in the outcome variables on a weekly level on average across all persons. We report 95% Bayesian credible intervals (CI) for all estimates, which describe the 95% probability range of the true estimate.

To test hypotheses 5 and 6, we employed cross‐level moderation analysis to test whether the five demands moderated the weekly within‐person relationships of planning behaviour with work engagement, unfinished tasks, rumination, and cognitive flexibility. We specified a single model in which the four random slopes of the effects of the planning manipulations on weekly unfinished tasks, work engagement, rumination, and cognitive flexibility were regressed on coordinating with others, planning of working times, planning of work places, structuring of work tasks, and predictability. To facilitate a meaningful interpretation of the results, the moderator variables were grand‐mean centered before the analysis. As we tested 20 moderation effects in total, we corrected them for multiple comparisons. The Bonferroni‐corrected α was .05/20 = .0025.

#### Control variables

We tested leadership position, tenure, age, education, working hours, the reported use of flexible time arrangements, and the amount of project work as potential control variables for the analyses. Age was the only variable that showed a significant relation to a dependent variable; it was therefore included as the only control variable in the final model. To reduce the complexity of the model and facilitate convergence, random slopes were set to be uncorrelated to the control variables. The study conclusions were robust including the control variable.

## RESULTS

We conducted multilevel confirmatory factor analyses (MCFAs) to test the construct validity of the study variables. In the first MCFA, we tested whether work engagement, unfinished tasks, rumination, cognitive flexibility, time‐management planning, and contingency planning represented empirically distinguishable constructs. All six variables were measured with self‐reported items at the same time point. For the hypothesized model with six separate factors on both analytical levels for all six variables, the MCFA showed a satisfactory fit, χ^2^ = 604.96, *df* = 274. The root mean square error of approximation (RMSEA) was .033, the comparative fit index (CFI) was .96, the Tucker–Lewis index (TLI) was .95, and the Akaike information criterium (AIC) was 61473.06. The six‐factor model had a better fit than the five‐factor model with the best fit, χ^2^ = 871.31, *df* = 284, RMSEA = .043, CFI = .93, TLI = .91, AIC = 61746.56, and then a one‐factor model, χ^2^ = 6658.19, *df* = 304, RMSEA = .138, CFI = .23, TLI = .13, AIC = 68451.22. These results suggest that the six variables represent empirically distinct constructs. With the second MCFAs, we examined the between‐person variables planning of working times, planning of work places, coordinating with others, structuring of work tasks, and unpredictability. For the hypothesized model with five separate factors on the between‐person level, the MCFA showed a very good fit χ^2^ = 161.73, *df* = 109, RMSEA = .021, CFI = .97, TLI = .96, AIC = 12,085.784. The five‐factor model had a better fit than the four‐factor model with the best fit, χ^2^ = 324.83, *df* = 113, RMSEA = .042, CFI = .86, TLI = .83, AIC = 12255.54, and then a one‐factor model χ^2^ = 1182.75, *df* = 119, RMSEA = .091, CFI = .29, TLI = .19, AIC = 13295.89. These results suggest that the five variables represent empirically distinct constructs.

We then investigated whether the planning manipulation was successful in changing participants' planning behaviour. We found that the planning manipulation showed significant effects on weekly time‐management planning, unstandardized estimate (γ) = .56, 95% CI [.40, .71], *R* ^2^ = .124 and on weekly contingency planning,^2^ γ = 1.07, 95% CI [.84, 1.27], *R* ^2^ = .187. This suggests that the planning manipulation was successful in increasing participants' weekly planning behaviour. Table 1 presents the descriptive statistics and variable intercorrelations.

**TABLE 1:** Means, standard deviations, and intercorrelations among study variables.

Variable	1	2	3	4	5	6	7	8	9	10	11	12
1. Planning manipulation	–	.05	**−.12**	**−.10**	**.11**	**.33**	**.46**	–	–	–	–	–
2. Work engagement	–	–	**−.18**	**−.33**	**.19**	**.25**	**.13**	–	–	–	–	–
3. Unfinished tasks	–	**−.47**	–	**.33**	−.09	−.06	**−.13**	–	–	–	–	–
4. Rumination	–	**−.45**	**.50**	–	−.03	−.09	−.05	–	–	–	–	–
5. Cognitive flexibility	–	**.40**	−.17	−.17	–	**.20**	**.28**	–	–	–	–	–
6. Time‐management planning	–	**.20**	−.17	.14	**.25**	–	.**51**	–	–	–	–	–
7. Contingency planning	–	**.22**	−.15	.15	**.40**	**.68**	–	–	–	–	–	–
8. Coordinating with others	–	**.36**	−.09	−.06	**.19**	.09	.12	–	–	–	–	–
9. Structuring of work tasks	–	**.18**	−.05	−.02	−.01	.15	−.02	.07	–	–	–	–
10. Planning of working times	–	.14	−.13	−.18	.02	−.004	−.01	.06	**.29**	–	–	–
11. Planning of working places	–	.12	.08	−.05	.14	.14	.13	.10	**.19**	**.21**	–	–
12. Predictability	–	**.28**	−**.30**	−.16	**.20**	.13	.09	.09	**.19**	.14	.06	–
*M*	.62	4.74	3.63	3.11	4.69	4.75	3.87	5.27	6.03	5.91	4.17	4.60
Within‐Person *SD*	.48	.90	1.29	1.20	.81	.92	1.20	–	–	–	–	–
Between‐Person *SD*	–	1.27	1.67	1.65	1.30	1.32	1.52	1.41	1.07	1.38	1.89	.99

*Note*: Correlations below the diagonal represent between‐person correlations (*N* = 208). Correlations above the diagonal represent within‐person correlations (*n* = 947). Due to the study design, the planning manipulation showed very low between‐person variance and did not allow us to estimate correlations on the between‐person level. All bold values *p* < .05.

Hypotheses 1 through 4 refer to the relationships of the planning manipulation with weekly work engagement, weekly unfinished tasks, weekly rumination, and weekly cognitive flexibility. The statistical model to test these hypotheses was first evaluated with regard to MCMC convergence. We increased the iterations to 250,000 until the trace plots showed visual convergence for all parameters. The final potential scale reduction factor was 1.003. The autocorrelation plots showed moderate autocorrelation for some parameters. However, since the trace plots and the potential scale reduction factor indicated that convergence was obtained, we concluded that the autocorrelation was acceptable (Depaoli & van de Schoot, 2017).

The results of the effects of the planning manipulation are outlined in Table 2. The planning manipulation was not found to be significantly related to weekly work engagement. Thus, hypothesis 1 was rejected. However, there was a negative relationship with weekly unfinished tasks, a negative relationship with weekly rumination, and a positive relationship with weekly cognitive flexibility, which supports hypotheses 2 through 4.

**TABLE 2:** Effects of the planning manipulation on work engagement, unfinished tasks, rumination and cognitive flexibility.

	Work engagement		Unfinished tasks		Rumination		Cognitive flexibility	
	Estimate	Bayesian 95% CI	Estimate	Bayesian 95% CI	Estimate	Bayesian 95% CI	Estimate	Bayesian 95% CI
Within‐person level								
Planning manipulation	.05	[−.11, .20]	−.29*	[−.51, −.06]	−.25*	[−.46, −.03]	.19*	[.05, .33]
*R* ^*2*^	.053*	[.03, .09]	.053*	[.03, .08]	.078*	[.05, .11]	.059*	[.04, .09]
Between‐person level								
Intercept	4.04*	[3.55, 4.52]	4.05*	[3.42, 4.68]	2.48*	[1.83, 3.12]	4.30*	[3.77, 4,84]
Age	.02*	[.01, .03]	−.01	[−.03, .01]	.02*	[.002, .04]	.01	[−.003, .02]

*Note*: Table shows unstandardized estimates. Point estimates are means. *R* ^2^ is within‐person level variance explained averaged across all persons. *N* = 208 individuals with 947 observations.

* *p* < .05.

The model to test the cross‐level moderation analyses hypotheses was first evaluated with regard to MCMC convergence. We increased the iterations to 500,000 until the trace plots showed visual convergence for all parameters. The final potential scale reduction factor was 1.003. The autocorrelation plots showed moderate autocorrelation for some parameters. However, since the trace plots and the potential scale reduction factor indicated that convergence was obtained, we concluded that the autocorrelation was acceptable (Depaoli & van de Schoot, 2017).

The results of the cross‐level moderation analyses are outlined in Table 3. We found no significant interaction effects for any of the four cognitive demands of flexible work. Thus, hypothesis 5 was rejected. Furthermore, we found no significant interaction effects of person‐level predictability on the effects of planning manipulation. Thus, hypothesis 6 was also rejected.

**TABLE 3:** Results of the cross‐level moderation analyses.

Parameter	Work engagement		Unfinished tasks		Rumination		Cognitive flexibility	
	Estimate	Bayesian 95% CI	Estimate	Bayesian 95% CI	Estimate	Bayesian 95% CI	Estimate	Bayesian 95% CI
Intercept	4.07*	[3.60, 4.53]	4.04*	[3.41, 4.67]	2.49*	[1.84, 3.15]	4.27*	[3.74, 4.80]
Age	.02*	[.004, .03]	−.01	[−.02, .01]	.02*	[.002, .04]	.01	[−.002, .03]
Planning manipulation	.05	[−.11, .20]	−.28*	[−.50, −.06]	−.25*	[−.46, −.03]	.19*	[.05, .33]
Coordinating with others	.20*	[.11, .30]	−.06	[−.19, .07]	−.04	[−.17, .10]	.12	[.003, .23]
Planning of working times	.05	[−.05, .16]	−.10	[−.24, .03]	−.11	[−.25, .04]	.002	[−.12, .12]
Planning of working places	.03	[−.05, .10]	.07	[−.02, .17]	.002	[−.10, .10]	.07	[−.02, .15]
Structuring of work tasks	.04	[−.10, .17]	.04	[−.14, .23]	.02	[−.17, .21]	−.09	[−.25, .06]
Predictability	.19*	[.05, .33]	−.30*	[−.48, −.12]	−.18	[−.38, .01]	.20*	[.04, .36]
PM × coordinating with others	−.11	[−.23, .002]	.07	[−.09, .23]	−.002	[−.16, .16]	−.06	[−.17, .05]
PM × planning of working times	−.01	[−.13, .12]	−.03	[−.21, .14]	−.08	[−.25, .09]	.04	[−.04, .11]
PM × planning of working places	.08	[−.01, .16]	−.07	[−.19, .04]	−.01	[−.13, .10]	−.01	[−.12, .10]
PM × structuring of work tasks	.08	[−.08, .24]	−.02	[−.25, .20]	−.20	[−.42, .02]	.02	[−.13, .16]
PM × predictability	.06	[−.11, .23]	.23	[−.01, .46]	−.06	[−.29, .18]	.02	[−.14, .17]

*Note*: Table shows unstandardized estimates. Point estimates are means. *N* = 201–203 and *n =* 926–930 observations.

Abbreviation: PM, planning manipulation.

* *p* < .05.

### Additional analyses

To gain further insight into the effects of planning behaviour, we conducted additional analyses. First, we checked for any potential effects of time by examining whether planning behaviour or outcome variable levels changed from week 1 to week 2. Time did not show significant effects on weekly time‐management planning, γ = .09, 95% CI [−.11, .29] or weekly contingency planning, γ = .06, 95% CI [−.18, .29]. Neither did we find any significant effects of time in the 2 weeks preceding the planning manipulation on weekly work engagement, γ = .09, 95% CI [−.11, .29], weekly unfinished tasks, γ = −.11, 95% CI [−.35, .15], or weekly cognitive flexibility, γ = .02, 95% CI [−.15, .19]. However, we did find a negative effect of time on weekly rumination, γ = −.49, 95% CI [−.76, −.21].

Second, we examined whether the outcomes increased with each subsequent completion of the planning manipulation, as participants may have needed time to adapt to the planning manipulation to learn how to plan effectively. Subsequent completion showed no effects on time‐management planning, γ = .06, 95% CI [−.01, .13], but we found a significant positive effect on contingency planning, γ = .18, 95% CI [.09, .26]. We did not find any significant effects of subsequent completion of the planning manipulation on weekly work engagement, γ = .01, 95% CI [−.06, .08], weekly unfinished tasks, γ = −.09, 95% CI [−.20, .01], weekly rumination, γ = −.03, 95% CI [−.12, .06], or weekly cognitive flexibility, γ = .07, 95% CI [−.001, .1].

Third, we investigated whether weekly time‐management planning and weekly contingency planning would mediate the effects of the planning manipulation on the four outcome variables. To do so, we included time‐management planning and contingency planning as within‐person mediator variables in a model with random intercepts. The results of the mediation analysis are outlined in Table 4. The analysis revealed that time‐management planning mediated a significant positive effect of the planning manipulation on work engagement. Contingency planning, but not time‐management planning, mediated significant effects of the planning manipulation on unfinished tasks and cognitive flexibility. For rumination, we found no significant direct or indirect effects.

**TABLE 4:** Results of mediation analysis.

	Work engagement		Unfinished task		Rumination		Cognitive flexibility	
	Estimate	Bayesian 95% CI	Estimate	Bayesian 95% CI	Estimate	Bayesian 95% CI	Estimate	Bayesian 95% CI
Within‐Level								
PM ➔ TMP	.61*	[.48, .74]	.61	[.48, .74]	.61	[.48, .74]	.61	[.48, .74]
PM ➔ CP	1.13*	[.98, 1.28]	1.13	[.98, 1.28]	1.13	[.98, 1.28]	1.13	[.98, 1.28]
PM ➔ Outcome	−.11	[−.26, .03]	−.17	[−.39, .05]	−.19*	[−.39, .02]	−.06	[−.19, .08]
TMP ➔ Outcome	.24*	[.15, .32]	.04	[−.09, .17]	−.09	[−.21, .03]	.07*	[−.01, .15]
CP ➔ Outcome	.02	[−.05, .10]	−.12*	[−.23, −.01]	.004	[−.10, .11]	.17*	[.10, .24]
Indirect effects								
PM ➔ TMP ➔ Outcome	.14*	[.08, .21]	.02	[−.06, .10]	−.05	[−.13, .02]	.04	[.01, .09]
PM ➔ CP ➔ Outcome	.03	[−.05, .11]	−.14*	[−.27, −.01]	.01	[−.11, .12]	.19*	[.12, .28]
Total indirect effect	.17*	[.09, .26]	−.12*	[−.23, .00]	−.05	[−.16, .06]	.24*	[.16, .32]
Total effect	.06	[−.08, .19]	−.29*	[−.47, −.10]	−.24*	[−.41, −.06]	.18*	[.06, .30]
Between‐Level								
Intercept	4.08*	[3.59, 4.58]	2.51*	[1.87, 3.15]	4.04*	[3.41, 4.66]	4.29*	[3.78, 4.81]
Age	.02*	[.004, .03]	−.01	[−.03, .01]	.02*	[.001, .03]	.01	[−.002, .03]

*Note*: Table shows unstandardized estimates. Point estimates are means. *N* = 201–203 and *n =* 926–930 observations.

Abbreviations: CP, contingency planning; PM, planning manipulation; TMP, time‐management planning.

* *p* < .05.

## DISCUSSION

The aim of this study was to examine the effects of a planning manipulation that combined different self‐regulation strategies in a real‐life work setting. Our results revealed that planning behaviour leads to fewer unfinished tasks and rumination and positively affects cognitive flexibility, but it does not have any effect on work engagement. Moreover, we did not find any significant moderation effects for cognitive demands of flexible work or predictability on the effects of planning behaviour.

### Theoretical implications

In line with our predictions, we found that engaging in planning behaviour at the beginning of the week was related to fewer unfinished tasks at the end of the week. This finding confirms the proposition of self‐regulation theory that planning behaviours support employees in finishing their tasks and achieving their goals. Our results complement the findings of previous observational studies (Parke et al., 2018; Tripoli, 1998), laboratory studies (Diefendorff & Lord, 2003), and studies on planning behaviour in areas other than work life, such as academic performance and education (Brandstätter et al., 2003; Britton & Tesser, 1991) or health‐related behaviours (Armitage, 2004; Sniehotta et al., 2006; Ziegelmann & Lippke, 2007). To the best of our knowledge, our study is the first to show such effects by manipulating planning behaviour in a naturalistic work setting.

As hypothesized, planning behaviour had positive effects on employees' weekly cognitive flexibility. This important evidence supports the notion that planning behaviour can foster more flexibility and adaptability in thinking, which means that employees can develop better perspective‐taking, problem solving, and innovative behaviours (Dennis & Vander Wal, 2010; Kohn & Schooler, 1983; Parker et al., 2008). Such cognitive benefits demonstrate that there are manifold ways in which planning behaviour can improve employees' performance (Frese & Zapf, 1994). These results may open a potential pathway to increase general levels of cognitive flexibility in employees. By regularly engaging in planning behaviour, cognitive flexibility may become automatized and transfer to other life domains (Frese & Zapf, 1994; Kohn & Schooler, 1983). Thus, our study yields much‐needed evidence that planning behaviour can leverage employee learning and personal development (Mumford et al., 2001; Wood et al., 2013).

In line with our Hypothesis 3, we found that planning behaviour had a negative effect on weekly rumination. This result is in line with earlier studies that found negative effects of planning behaviour on rumination; it also reveals that planning behaviour could be an important intervention strategy for employees who struggle with rumination (Masicampo & Baumeister, 2011; Smit, 2016; Smit & Barber, 2016). Setting goals and developing specific plans seems to help individuals mentally disengage from tasks when necessary. The negative effect on rumination may have resulted from a decrease in unfinished tasks throughout the week (Syrek et al., 2017), but our study design did not allow us to test this hypothesis. Future research should seek to illuminate the processes that link planning behaviour to reduced rumination.

Contrary to our expectations, we found no evidence for the positive effects of planning behaviour on weekly work engagement. This result is in contrast to Parke et al.'s (2018) earlier study. However, their study was observational; therefore, other variables could have confounded the relationships. Since we manipulated planning behaviour, our study provides a stronger test on the effects of planning behaviour on work engagement. Considering that other studies that examined related hypotheses did not find motivational benefits for planning behaviour in a work context (Casper & Sonnentag, 2020; Slaven & Totterdell, 1993), evidence for motivational benefits of planning behaviour at work seems to be mixed. The time horizon and the regularity of the manipulated planning behaviour may explain these mixed findings. Parke et al.'s (2018) study examined daily planning behaviour, which has a much shorter time horizon and higher regularity than weekly planning behaviour. Goals in weekly planning are temporally further away, which could weaken its motivational effects (Steel & König, 2006) or lead employees to choose tasks that are less motivating (Bakker & Oerlemans, 2019). Compared to daily planning, planning once a week may not be sufficiently frequent to provide employees with necessary information on their goal progress (Ahmetoglu et al., 2021; Johnson et al., 2013). The temporal dynamic regarding planning behaviour has not received concerted attention in the literature (e.g., Claessens et al., 2007; Fried & Slowik, 2004; Lord et al., 2010; Sonnentag, 2012), however, and more theoretical development and empirical research on the role of time in planning behaviours seem necessary. More specifically, future research should examine whether and how the motivational benefits of planning behaviour are contingent on the time horizon and regularity of planning behaviour.

In our model, 5.3% of the variance in weekly unfinished tasks, 7.8% of the variance in weekly rumination, and 5.9% of the variance in weekly cognitive flexibility were explained on average across all participants. This corresponds to correlations of *r* = .25 to *r* = .30. Various issues must be considered when interpreting these effect sizes (Bakker et al., 2019). First, intervention studies aiming to change similar outcomes at work found similar effect sizes (Carolan et al., 2017; Donaldson et al., 2019; Karabinski et al., 2021; Knight et al., 2017; Vîrgă et al., 2021). Effects were smaller when the intervention dosage was less than 4 h, when addressing individuals rather than groups and when delivered online, as was the case in our study. Thus, the effect sizes reported here are not unusual.

Second, effect sizes should be compared to the time and effort invested (Bakker et al., 2019). Considering that the median time participants invested to complete the planning manipulation were less than 8 min, the cost‐effectiveness ratio still appears good; the effect sizes could still represent practically relevant effects of planning behaviour (e.g., when more time is invested). Future research should examine whether repeated or more detailed planning behaviour throughout the week shows stronger effects. It would also be useful to examine when the additional benefits of more planning behaviour begin to decrease.

The specific pattern of beneficial effects on weekly unfinished tasks and cognitive flexibility in combination with the non‐significant results regarding weekly work engagement warrants consideration. One discussion in the self‐regulation literature focuses on the question of whether planning behaviour improves goal achievement via motivational benefits (e.g., fostering and facilitating higher investment of effort) or via intellectual benefits (Diefendorff & Lord, 2003; Gollwitzer, 1996). The improved goal achievement revealed in the current study does not seem to come from motivational benefits, as this should manifest in higher weekly work engagement. Instead, the positive effects on weekly cognitive flexibility support the notion that planning behaviour improves goal achievement via better strategies and an improved understanding of the task (Diefendorff & Lord, 2003; Frese & Zapf, 1994). However, our data are not suited to drawing conclusions on this matter as we have not examined the mediating variables theorized here.

Additional analyses allowed us to gain further insight into our findings. Notably, these analyses showed that subsequent completion of the planning manipulation positively affected contingency planning which suggests that employees first had to learn how to implement the strategy. Actively developing alternative plans may be used less often than other strategies and is likely also more complex to execute. Higher levels of contingency planning were subsequently associated with fewer unfinished tasks and higher cognitive flexibility. With regard to finishing tasks and cognitive flexibility, the benefits of planning behaviour seem to result from thinking about potential obstacles and alternative plans. Such strategies could encourage employees to build more elaborate mental models of how to execute a task (Frese & Zapf, 1994; Zacher & Frese, 2018). This finding further supports the proposition that the benefits of planning behaviour are more cognitive and strategic than motivational and stemming from an increased investment of effort.

Furthermore, we found evidence for an indirect association of planning manipulation with work engagement via time‐management planning, which is in line with the findings of Parke et al. (2018). However, due to the non‐significant total effect, the effects of planning behaviour on work engagement appear to be less robust. As discussed above, they may be more complex and depend on additional variables, such as the regularity and time frame of planning behaviour.

In sum, the mediation analyses indicate that different outcomes were affected by different parts of planning behaviour. This shows that combining various self‐regulation strategies increases the chances of benefiting a wider range of outcomes. Therefore, a promising avenue for future research would be to clarify which strategies are important to combine and whether their effects are additive or interactive. Once clarified, self‐regulation strategies could be better tailored to the outcomes one intends to affect.

The expected moderation effects for the cognitive demands of flexible work and predictability were not supported in our study. Though our sample size was relatively large, cross‐level interaction effects can be difficult to identify (Arend & Schäfer, 2019). The non‐significant interaction effects found in this work could therefore result from a lack of power in our study design. Moreover, an analysis of the variability of the person‐level demands in our sample demonstrated that planning of working times and structuring of work tasks were strongly left‐skewed and had high kurtosis. Planning of working times showed a skewness of −1.70 and a kurtosis of 2.90 and structuring of work tasks a skewness of −1.60 and a kurtosis of 3.18. Thus, range restrictions could have biased the analysis of moderation effects for these two demands. To detect any potential interaction effects, future research should ensure that recruited study samples have high variability in person‐level demands.

Planning of work places, coordinating with others, and predictability were not strongly skewed, nor did they show strong peaks or flatness, which suggests that lack of variability should not have been an issue for these demands. The effects of the planning intervention seem to be independent of the level of these demands. An explanation for the non‐significant interaction effects could be that the manipulated planning behaviours were overly focused on formal and linear planning techniques and were therefore not sufficiently tailored for the planning of work places, coordinating with others, and predictability. More opportunistic and incremental planning methods with shorter planning intervals, such as agile methods with daily stand‐ups, may be more useful for particularly flexible and unpredictable work environments (Fernandez & Fernandez, 2008; Hacker, 2003; Zwikael & Gilchrist, 2021). Further, collective rather than individual planning behaviour may be more important, as collaboration is often a central part of jobs with such demands (Grant & Parker, 2009; Prem et al., 2021); collective planning may trigger crucial variables such as social support and information flow (Kramer et al., 2013; Tessem, 2014). To further advance the application of self‐regulation theory to the world of work, future research should examine whether and how the benefits of self‐regulation strategies can be strengthened through adaptation to different work contexts (Lord et al., 2010).

### Practical implications

Our results suggest that employees should be encouraged to set work goals for one week at a time, create a detailed plan for how they will achieve those goals, think about obstacles, and develop appropriate alternative plans. Employee planning behaviour can be facilitated by setting specific and challenging goals, specifying clear prioritization, ensuring employees possess the necessary expertise and information, and providing useful time‐management tools (Ahmetoglu et al., 2021; Locke & Latham, 2002; Mumford et al., 2001). Organizations and supervisors should encourage employees to specifically consider potential hindrances and develop alternative plans, as these strategies seem to be less common among employees and they may benefit performance the most. Planning behaviour can be improved by reflecting on the success of planning processes after projects have been finalized to identify where planning went wrong (Frese & Zapf, 1994; Mumford et al., 2015). Further, our planning manipulation could be adapted as a possible training tool to guide employees towards the habit of planning (Claessens et al., 2007).

### Limitations and avenues for future research

Our study design allowed us to overcome some of the methodological shortcomings of earlier studies, but it was not without limitations. Since all participants took part in 2 weeks of the study without planning manipulation and 5 weeks with planning manipulation, we cannot rule out the possibility that participant performance may have improved in the variables over time regardless of the planning manipulation. Such effects could occur by inducing certain changes in variables through repeated questioning (Gabriel et al., 2019). Further, additional analysis showed no evidence for the effects of time on planning behaviour, weekly work engagement, unfinished tasks, and cognitive flexibility in the 2 weeks preceding the planning. There was, however, a significant negative effect of time on rumination. Future research should thus seek to replicate the beneficial effects of planning behaviour on rumination. Finally, our study design did not allow us to rule out improvements in the outcome variables due to participant expectations (Green et al., 2014). To address these issues, future research could use wait‐list control groups and active control groups, which would allow the effects of time to be disentangled from the effects of planning manipulation.

It is also important to note that our design only allows for an explanation of the variability between the 2‐week versus 5‐week conditions. It cannot explain variability within the first 2 weeks or the subsequent 5 weeks. This is because the planning manipulation was the only predictor and it did not change in the first 2 weeks or in the subsequent 5 weeks. Nonetheless, the weekly diary design is a study strength, as it would have reduced the influence of random fluctuations in our dependent variables and considerably improved the validity of the measurements.

## AUTHOR CONTRIBUTIONS

**Lars Uhlig:** Conceptualization; data curation; formal analysis; investigation; methodology; project administration; software; visualization; writing – original draft; writing – review and editing. **Vera Baumgartner:** Conceptualization; investigation; methodology; project administration; resources; visualization; writing – review and editing. **Roman Prem:** Conceptualization; formal analysis; methodology; software; validation; writing – review and editing. **Katja Siestrup:** Investigation; resources; writing – review and editing. **Christian Korunka:** Conceptualization; funding acquisition; project administration; resources; software; supervision; writing – review and editing. **Bettina Kubicek:** Conceptualization; funding acquisition; project administration; resources; software; supervision; writing – review and editing.

## FUNDING INFORMATION

This work was supported by the Austrian Science Fund (FWF): P29408‐G29.

## CONFLICT OF INTEREST STATEMENT

The authors report no potential conflict of interest.

## Supporting information


Appendix S1

## Data Availability

The data used in this research is not readily available, as the participants have not been asked for consent to share the data publicly. Requests to gain access to the data should be directed to the corresponding author.

## Footnotes

^1^
Further details are provided in the Appendix S1.

^2^
Due to a programming error in the online questionnaire, the first 20 participants were not asked the items regarding time management planning and contingency planning. Thus, the sample size for the results of the manipulation check is reduced to *N* = 175 individuals and *N* = 784 observations.
