# Supplementary material for: A field experiment on the effects of weekly planning behaviour on work engagement, unfinished tasks, rumination, and cognitive flexibility
Source: J Occup Organ Psychol. 2023 Feb 17;96(3):575–98. doi: 10.1111/joop.12430 (PMC10952538; doi:10.1111/joop.12430)
Supplement: Supplementary file 1 — Appendix S1 [file JOOP-96-575-s001.docx]

# **Supplementary materials for:**

***A field experiment on the effects of weekly planning behavior on work engagement, unfinished tasks, rumination, and cognitive flexibility***

An analysis of differences between the student-recruited sample and the sample recruited via the newsletter and the IT company using *t*-tests can be found in Table S1. As we tested 15 group differences in total, we corrected for multiple comparisons. The Bonferroni-corrected α was .05/15 = .0033. The comparison showed that the participants in the sample recruited via the newsletter and the IT company were of younger age than participants in the student-recruited sample. Mann-Whitney U tests showed no significant difference between both samples regarding the educational level (*U* = 4862, *p* = .86) and leadership position (*U* = 5244, *p* = .20). There was also no significant association between gender and sample type, χ^2^(1, 200) *=* .02, *p* = .90.

| Table S1. Sample comparison | | | | | |
| --- | --- | --- | --- | --- | --- |
|  | Merged sample of newsletter and IT company | | Student-recruited sample | |  |
|  | *M* | *SD* | *M* | *SD* | *t-*test |
| Age | 34.43 | 10.49 | 42.00 | 11.63 | 4.47*** |
| Tenure | 5.59 | 5.23 | 4.28 | 5.09 | 1.74 |
| Working hours | 39.42 | 10.06 | 37.66 | 11.44 | 1.11 |
| Work engagement | 4.78 | 1.04 | 4.60 | 1.01 | 1.25 |
| Unfinished tasks | 3.90 | 1.31 | 3.69 | 1.34 | 1.12 |
| Rumination | 3.42 | 1.49 | 3.04 | 1.21 | 2.04 |
| Cognitive flexibility | 4.75 | 1.12 | 4.67 | 1.08 | 0.47 |
| Time-management planning | 4.57 | 1.27 | 4.67 | 1.09 | -0.57 |
| Contingency planning | 3.47 | 1.24 | 3.82 | 1.16 | -1.82 |
| Coordinating with others | 5.36 | 1.30 | 5.20 | 1.48 | 0.73 |
| Planning of working times | 6.00 | 1.45 | 5.84 | 1.34 | 0.80 |
| Planning of work places | 3.71 | 1.96 | 4.46 | 1.81 | -2.77 |
| Structuring work tasks | 6.19 | 0.98 | 5.92 | 1.12 | 1.71 |
| Predictability | 4.41 | 1.02 | 4.71 | 0.97 | -2.09 |
| *Note*. Newsletter recruited and organizational sample *N* = 83; Student-recruited sample *N* = 125; Bonferroni-corrected α = .0033. * *p* < .05 | | | | | |
